# Oral cannabidiol did not impair learning and memory in healthy adults

**DOI:** 10.1186/s42238-025-00262-2

**Published:** 2025-01-23

**Authors:** Hanna H. Gebregzi, Joanna S. Zeiger, Jeffrey P. Smith, Libby Stuyt, Luann Cullen, Jim Carsella, Daniel C. Rogers, Jordan Lafebre, Jennah Knalfec, Alfredo Vargas, Moussa M. Diawara

**Affiliations:** 1 Department of Biology, Colorado State University Pueblo, 2200 Bonforte Blvd, Pueblo, CO 81001 USA; 2 Clinical Research Organization, ICON PLC, 8307 Gault Lane, San Antonio, TX 78209 USA; 3 Cann Research Foundation, 3996 Savannah Ct, Boulder, CO 80301 USA; 4 Circle Program, Colorado Mental Health Institute at Pueblo, 1600 W 24th Street, Pueblo, CO 81003 USA; 5 Cullen Regenerative Medicine, Naturopathic Medicine, 112 W D St, Pueblo, CO 81003 USA; 6 Department of Chemistry, Colorado State University Pueblo, 2200 Bonforte Blvd, Pueblo, CO 81001 USA

**Keywords:** CBD, THC, L&M, MOCA, Prose recall, Logical Memory Subtest, WMS, RAVLT-R, PI ratio, RI ratio

## Abstract

**Background:**

The effect of oral Cannabidiol (CBD) on interference during learning and memory (L&M) in healthy human volunteers has not been studied.

**Method:**

A two-arm crossover, randomized, double-blind, placebo-controlled trial was conducted at Colorado State University Pueblo (CSU Pueblo) to evaluate the effects of 246 mg oral CBD on L&M in healthy adults. Among 57 healthy volunteers enrolled, 35 were included in the analyses. For assessment of L&M, Montreal Cognitive Assessment (MOCA) was used to evaluate verbal baseline cognitive function; RAVLT-R tests (List A and List B recalls, Proactive and Retroactive Interference ratios, and Forgetting Speed ratio) were used to evaluate verbal declarative memory; and total prose recall was used to evaluate verbal logical memory. Linear Mixed Models with Bonferroni Corrections were used to compare L&M results between primary outcomes (CBD vs. placebo) and secondary demographic outcomes, with a two-tailed statistical significance of *P* < 0.05.

**Results:**

CBD administration did not affect any of the dependent variables measured compared to the placebo group. There were no effects of THC, history of CBD use, or sex on CBD’s modulation of L&M. However, a highly significant interaction effect between treatment groups (CBD vs. placebo) and age of subjects was observed for the PI ratio (*P* = 0.008; *n* = 35).

**Conclusions:**

The results of this study suggest that administration of oral CBD alone does not significantly impair L&M in healthy adults. However, age might influence CBD related modulation of proactive interference during human L&M. Future research involving a larger group of older adults is needed to confirm this potential effect.

**Trial registration:**

The study was approved by the CSU Pueblo IRB, conducted in accordance with the Declaration of Helsinki, and registered with ClinicalTrials.gov (NCT06074172).

**Supplementary Information:**

The online version contains supplementary material available at 10.1186/s42238-025-00262-2.

## Background

Tetrahydrocannabinol (THC) and cannabidiol (CBD) are the two primary cannabinoids present in the cannabis plant. Studies have suggested that the use of THC, a psychogenic compound, may cause impairment with working memory (D’Souza et al. 2004; Hart et al. 2010) as well as verbal memory (Hart et al. 2001; Curran et al. 2002; D’Souza et al. 2004; D’Souza et al. 2005; Hunault et al. 2009) in a dose-dependent manner (Bolla et al. 2002; Curran et al. 2002; D’ Souza et al. 2004). In contrast, CBD has been reported to ameliorate the impairment that THC causes on verbal and working learning and memory (L&M) (Nicholson et al. 2004; Bhattacharyya et al. 2010; Morgan et al. 2010, 2012; Englund et al. 2013). Studies involving animal models also suggest the modulation of L&M by CBD. For example, CBD attenuated the effects of THC during visuospatial associative memory studies in adult male rhesus monkeys (Wright et al. 2013). Studies have also shown modulatory effects of CBD on fear L&M in animal models (Simone et al. 2015; Uhernik et al. 2018).

CBD exerts its effect on L&M through modulation of receptors of the endocannabinoid system. Cannabinoids are key modulators of synaptic function as they bind to CB1 and CB2 G-protein coupled receptors (Wilson et al. 2002; Castillo et al. 2012). These receptors are heavily expressed in the brain (Ford et al. 2021; Wilson et al. 2002) and have been studied to understand their role in healthy and pathological neuronal signaling (Scotter et al. 2010; Castillo et al. 2012; Busquets-Garcia et al. 2018; Ford et al. 2021). In vitro studies involving human or rat cells found CBD to be a negative allosteric modulator of the CB1 receptor (Petitet et al. 1998; Thomas et al. 2007; Laprairie et al. 2015; Tham et al. 2019), suggesting that CBD’s negative effects on the CB1 receptor could be a mechanism for enhanced synaptic plasticity. Activation of the CB1 receptor slows down synaptic transmission by inhibiting the release of neurotransmitters (Szabo et al. 2005). Thus, CBD has the potential to positively modulate learning through its negative effects on the CB1 receptor. CBD was also reported to prevent the progression of memory deficits in Alzheimer’s transgenic mice (Cheng et al. 2014). These combined findings point to CBD as a possible memory-enhancing agent.

To the authors’ knowledge no previous studies had examined the effect of oral CBD on L&M in healthy adults using Rey Auditory Verbal Learning Task-Revised (RAVLT-R) assessment, with the evaluation of Retroactive Interference ratio, Proactive Interference ratio, immediate and delayed prose recall as endpoints for analysis. There was a study conducted to investigate the effect of vaping CBD on verbal episodic memory in healthy young subjects (Hotz et al. 2021). That previous study found that CBD had positive effects on learning and memory. However, the study used vaping CBD e-liquid (0.25 ml, 5% CBD, 12.5 mg CBD), and the variable measured was a 20-minute short delay verbal memory recall.

Moreover, no studies that we are aware of have been done to date evaluating the effect of oral CBD on interference during learning in a healthy adult population. We hypothesized that CBD would be a positive modulator of human L&M through a mechanism of diminished retroactive and proactive interference. The objective of this study was to determine whether CBD exhibits enhancing effects on L&M through diminished Retroactive and Proactive Interference, and how this modulation might be influenced by demographic factors such as age, sex, history of CBD use, and marijuana use.

## Methods

### Design and participants

**Fig. 1:**
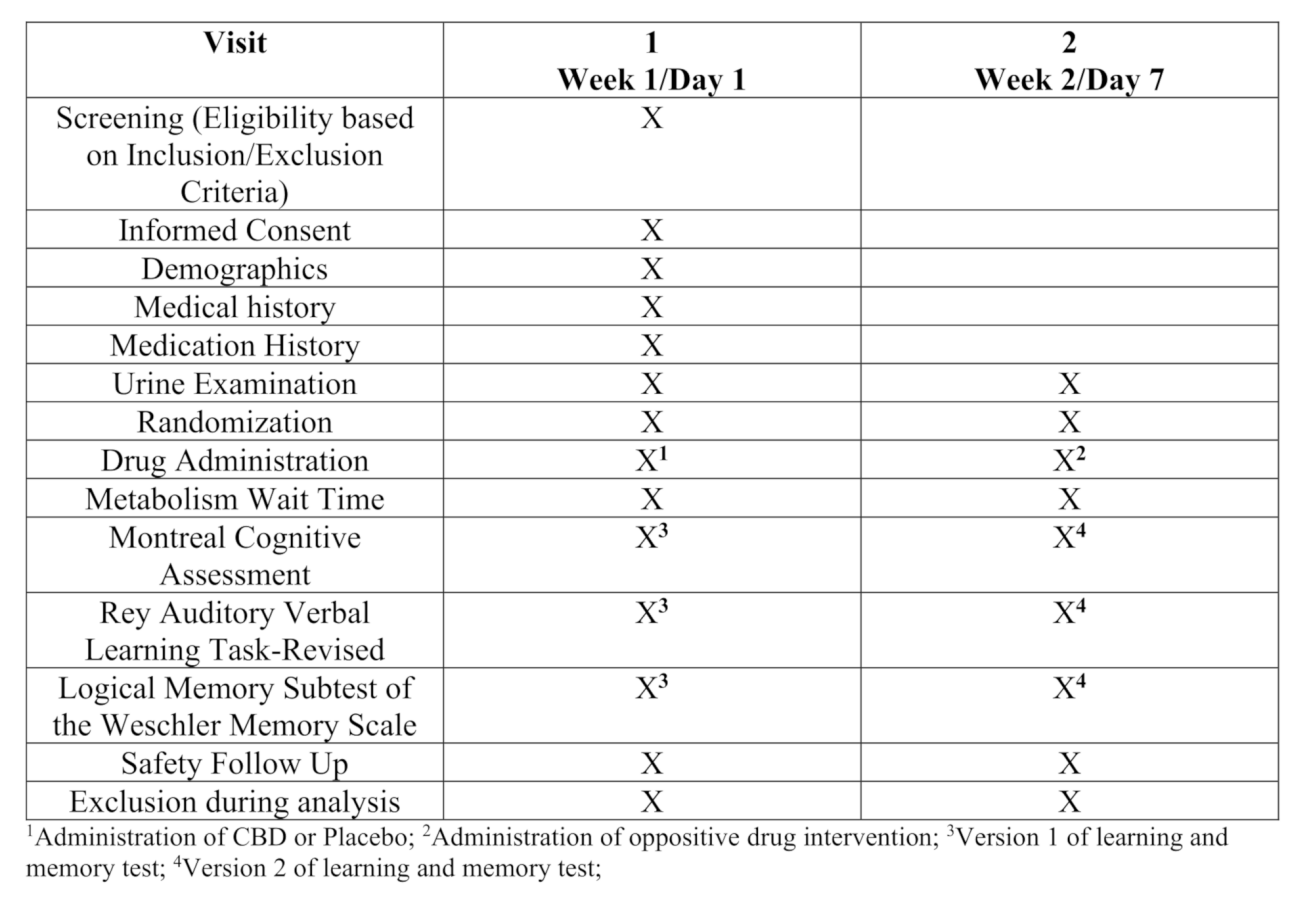
Flow-chart: Clinical Trial Activities

A randomized, two-arm crossover trial (Fig. 1) was conducted between Jan-Mar 2020 and Sep-Nov 2020 by recruiting healthy volunteers from Colorado State University Pueblo (CSU Pueblo) and the greater Pueblo community via posted advertisements, local newspapers, and by word of mouth. During screening, three participants were excluded; these included one who was pregnant, one who didn’t speak English fluently, and the third one who had severe hearing problems. A total of 57 participants were enrolled into the study; they were all over 18 years of age and there was no age limit used during the study. All participants signed an informed consent and filled out a demographic questionnaire, where they provided information about their age, ethnicity, sex, education, frequency of coffee consumption, and history of drug use (marijuana use and nicotine use). Participants self-reported if THC and/or CBD were used daily, once weekly, two to five times weekly, one to three times monthly, or never. Participants were tested during the urine analysis for use of THC and other drugs, as detailed later. To test for potential CBD-THC interactions, participants were included in the trial even if they tested positive for urine THC. Participants were not asked to abstain from using cigarettes, coffee, or medications. Additionally, participants were instructed to be consistent with their daily routines (e.g., sleep, activities, food and beverages) between the two study visits. The study was approved by the CSU Pueblo IRB, conducted in accordance with the Declaration of Helsinki and registered with ClinicalTrials.gov (NCT06074172).

The sample size was initially planned to include 200 participants, based on the population size of Pueblo, Colorado. However, the study was disrupted for six months due to COVID-19-related social distancing and the subsequent university closure. The number of participants assessed for eligibility was scaled down to 60, in proportion to the university community size. This number of subjects for a crossover design is still consistent with previous parallel or crossover clinical trials designed to examine the effects of oral CBD administered alone (Boggs et al. 2018) or CBD interaction with THC (Zuardi et al. 1982; Nicholson et al. 2004; Bhattacharyya et al. 2010; Zamarripa et al. 2023).

During each of the two visits of this study, participants were administered three press pills of CBD (82 mg each; total of 246 mg) or three pills placebo and were instructed to chew the tablets and keep them under their tongue for 2 min prior to swallowing, to allow for pseudo-sublingual absorption. Pseudo-sublingual administration was used during the current study because this form of exposure has been reported to allow for better delivery of CBD (Millar et al. 2020). The CBD and placebo press pills were obtained from Steve’s Goods CBD (Longmont, Colorado). They were prepared to look identical; they were all white in color and the same size. A one-week washout period was used between visits 1 and 2. On the second visit, participants received the opposite drug from their first visit. Some participants were not able to complete visit 2 one week later due to university closure and social distancing for six months. Therefore, the clinical trials were conducted from January-March 2020 and from September-November 2020. The 246 mg dose was used because previous studies suggest that this dose is sufficient to facilitate cognitive effects in healthy human subjects (Linares et al. 2019; Solowij et al. 2018).

Participants during this study were administered CBD or placebo and tested 2 h later for L&M. Participants completed the demographics questionnaire during the 2 h wait time. The 2 h waiting period is supported by studies that showed that the time frame of 1–2.5 h was sufficient to observe the effect of oral CBD alone (Crippa et al. 2004; Bhattacharyya et al. 2010; Patrician et al. 2019; Cherniakov et al. 2017) or CBD administered with THC (Fusar-Poli et al. 2009; Bhattacharyya et al. 2010). Research also shows Cmax for oral CBD alone to be 1–3 h (Cherniakov et al. 2017; Patrician et al. 2019; Britch et al. 2021; Hosseini et al. 2021), and the same time period was observed for the CBD/THC cocktail Cmax (Zamarripa et al. 2023).

### Randomization and blinding

Subjects were randomized to receive CBD or placebo using block randomization (each block of 4 containing two CBD and two placebo conditions). Generation of the randomization list was performed by the principal investigator (PI), who took precautions to prevent his own unblinding of subjects, assessors, and data analysts. The PI was not involved in data collection nor analysis to prevent any bias. Subjects were administered either CBD or placebo pills under the supervision of the unblinded PI. Participants and data collectors were all blinded to the study intervention groups.

### Chemical analysis of CBD and placebo pills

CBD and placebo press pills were analyzed for purity, THC content, heavy metals, bacteria, and pesticides. The chemical analyses were conducted in the chemistry department at CSU Pueblo and by Botanacor, an independent lab in Denver, CO. Both laboratories found the CBD pills to be 99.98% pure; no THC, heavy metals, bacteria, or pesticides were detected.

### Cognitive assessments

Participants completed the Montreal Cognitive Assessment (MOCA), the Rey Auditory Verbal Learning Task-Revised (RAVLT-R), and the Logical Memory Subtest of the Wechsler Memory Scale (or prose recall). The duration of the L&M assessments was approximately 30 min. The participants completed a different version of the cognitive assessment battery administered during each of two visits (1 and 2); the two versions were equal in difficulty.

#### MOCA

The MOCA is a written, non-invasive assessment of basal cognitive function. MOCA was used to evaluate verbal baseline cognitive function. MOCA was administered by a tester and took 5–10 min to complete. MOCA assessment of visuospatial and executive function, memory, and abstraction was done as previously described (Mast 2010).

#### RAVLT-R

To evaluate verbal declarative memory, two different versions of the RAVLT-R test were utilized (Solowij et al. 2011; Meier et al. 2012; Becker et al. 2014; Khosravi Fard et al. 2016). Participants were instructed to listen to a pre-recorded list of 15 words (RAVLT-R List A) and then asked to recall List A in five different trials (List A trials A1, A2, A3, A4, and A5) with the words repeated to them a few seconds after each trial. Participants were scored for the number of correctly repeated words for each trial. Afterwards, the participants were instructed to listen to another pre-recorded list of 15 words (RAVLT-R List B), asked to recall List B once, and scored for the number of correctly repeated words. Participants were then immediately asked to recall List A (List A trial 6), and their responses were scored. Lists A and B were retrieved from the Rey Auditory Verbal Learning Test-Revised handbook (Schmidt et al. 1996). Participants were distracted with the prose recall test (described below). Following prose recall, participants were asked to complete a delayed recall of List A (List A trial 7), and scored for the number of correctly repeated words.

Two types of interference were quantified from the RAVLT-R: (1) Proactive Interference (PI) ratio = B/A1, where B is the number of words recalled from List B (distractor list), and A1 is the number of words recalled from List A on the 1st trial; (2) Retroactive Interference (RI) ratio = A6/A5, where A6 is the number of words recalled from List A after the distractor List B, and A5 is the number of words recalled from List A on the 5th trial. Assessing Proactive Interference allows for the determination of how learning old material impacts learning of new information; this was assessed in this study by determining how List B recall was impacted after List A recall. Retroactive Interference is the impact of new information on previously learned information, this was measured by examining how immediate recall of List A was impacted after List B recall. The effect of forgetting was quantified from RAVLT-R using the equation Forgetting Speed (FS) = A7/A6, where A7 is the number of words recalled from List A after the prose recall delay, and A6 is the number of words recalled from List A after the distractor List B. All these equations were previously utilized to evaluate the PI, RI, and FS ratios (Magalhães et al. 2012), and the ratios have been used in other L&M studies (Geffen et al. 1990; Vanderploeg et al. 2001; Kramer et al. 1991; Numan et al. 2000; Torres et al. 2001; Malloy-Diniz et al. 2007; Magalhães et al. 2012; Frith et al. 2018).

#### Prose Recall

To evaluate verbal logical or episodic memory, the Logical Memory Subtest of the Wechsler Memory Scale (also referred to as total prose recall) was used (Curran et al. 2002; Morgan et al. 2010; Hindocha et al. 2018). Participants were instructed to listen to a pre-recorded short prose story and asked to immediately repeat the story word for word. Participants were given one point for each word correctly repeated. Each response was compared to acceptable responses of the Wechsler Memory Scale-Revised Manual (Wechsler 1987). Scores for each correctly repeated word were added with a total possible score of 25. Participants were then asked to complete the delayed recall of List A of the RAVLT-R assessment, as described above. Afterwards, participants were asked to repeat the short story again. The two scores for the short story repeats were compiled to obtain the total prose recall score. The short story was retrieved from the same manual (Wechsler 1987).

### Urine analysis

Chemical analyses of urine samples were done using the 12 Panel CLIA WAIVED Drug Test Cup Kit (Lot #: D1910087; Carlsbad, Ca) which screened for the presence or absence of Amphetamine, Barbiturates, Benzodiazepines, Ecstasy, Buprenorphine, Cocaine, Methadone, Methamphetamines, Morphine, Oxycodone, Phencyclidine and THC. Except for THC, the presence of any other drugs was exclusionary.

### Statistical analysis

**Fig. 2:**
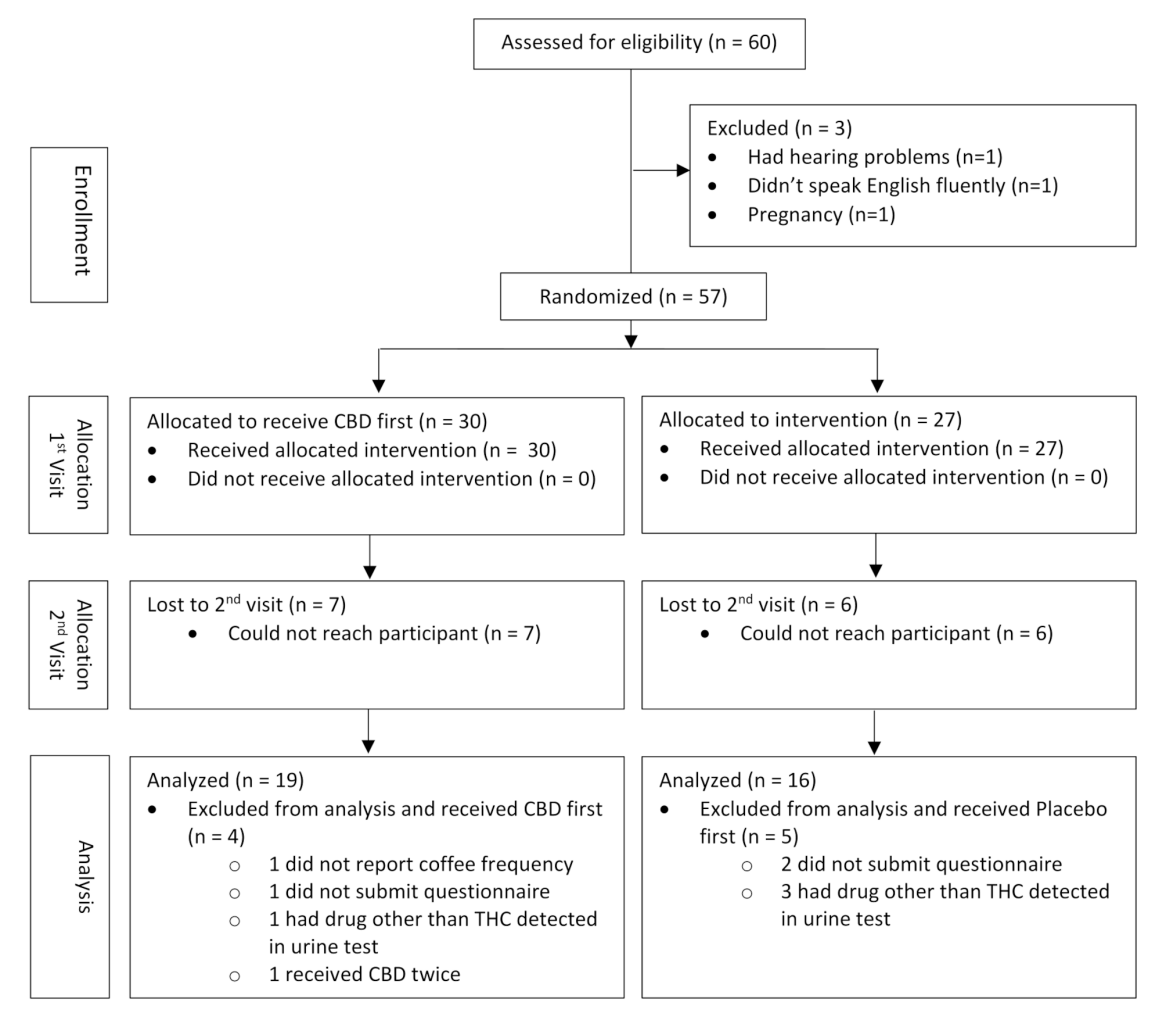
Consort diagram of the process through study phases

Only 35 of the 57 participants recruited were included in the final analysis; the others were excluded for reasons provided in Fig. 2. All statistical analyses were performed using SPSS (Version 28.0.1.1). Compound symmetry was the covariance structure used because it was determined to be the best fit for our Linear Mixed Models (LMM). Compound symmetry is the structure required for split-plot designs and the variances are homogeneous. This covariance structure has been used in other verbal memory test studies (Wang et al. 2023). With compound symmetry, it is assumed that the correlation between two measurements is constant regardless of how far apart the measurements are. Associations were examined using LMM with Bonferroni Corrections. Two-tailed *P* < 0.05 was considered statistically significant. Each LMM included the following fixed effects: treatment factor (CBD vs. placebo), and the following demographic covariates: age, history of CBD use, sex, and urine THC. The fixed effects also included the following interactions: treatment*age, treatment*sex, treatment*history of CBD use, and treatment*urine THC. Subject IDs were included as random effects.

The dependent variables used to compare the effects of the treatment groups (CBD vs. placebo) on L&M were our primary outcomes. The dependent variables used to examine the effects of demographic factors (urine THC, history of CBD use, sex and age) on L&M and the interactions between treatments and demographics were the trial secondary outcomes. The seven dependent variables were MOCA, Sum of List A trials 1–5, List B, Proactive Interference (PI) ratio, Retroactive Interference (RI) ratio, Forgetting Speed (FS) ratio, and Total prose recall (Table 1).

**Table 1:** Description of the Rey Auditory Verbal Learning Task-Revised (RAVLT-R) and prose recall measurements used for analyses (*n* = 35)

RAVLT-*R*	Description
Trial A1	Number of words recalled from list A on the 1st trial
Trial A2	Number of words recalled from list A on the 2nd trial
Trial A3	Number of words recalled from list A on the 3rd trial
Trial A4	Number of words recalled from list A on the 4th trial
Trial A5	Number of words recalled from list A on the 5th trial
**Sum of Trials A1-5**	**Sum of words recalled from list A across the 5 trials**
**Trial B**	**Number of words recalled from List B (distractor list)**
Trial A6	Number of words recalled from list A after the distractor list
Trial A7	Number of words recalled from list A after a 30-min delay
**PI Ratio**	**Proactive Interference ratio (trial B1/A1)**
**RI Ratio**	**Retroactive Interference ratio (trial A6/A5)**
**FS Ratio**	**Forgetting Speed ratio (trial A7/A6)**
**PROSE RECALL**	**Description**
Immediate	Number of items immediately recalled from the story
Delay	Number of items recalled from the story after a 30-min delay
**Total**	**Sum of items recalled from Immediate and Delay Prose Recall**

*For both Versions of the assessments, participants were instructed to listen to a list of 15 words (List A) read to them. They were then asked to recall List A in five different trials, with the words repeated to them after each trial. Participants were scored for the number of correctly repeated words for each trial. Afterwards, the participants were instructed to listen to another list of 15 words (List B), asked to recall List B once, and scored for the number of correctly repeated words. Participants were then immediately asked to recall List A, and their responses were scored. Participants were distracted with a prose recall test, by reading to them a short story and asking them to repeat the story word-for-word. Following the prose recall, participants were asked to complete a Delayed recall of List A. They were scored for the number of correctly repeated words

## Results

### Participant demographics

Information regarding the demographics of study participants is provided in Table 2. Among the 35 study participants retained for the analyses, 19 were female (54%) and the majority were between the ages of 18–27 (71.4%). The age of participants ranged from 18 to 64, with only four participants over 60. Most completed some college (49/2%) and 18 (51/4%) of the subjects self-identified as Caucasian.  Thirty seven percent of subjects reported daily coffee drinking while 28.6% reported they never drink coffee, 71.4% reported no CBD use, 85.7% reported they did not use cigarettes, and 54.3% had a negative urine THC result.

**Table 2:** Demographic data of the trial participants (*n* = 35)

Demographic Factor		*N* (%)
Sex	Female	19 (54)
	Male	16 (46)
Age	18–25	23 (66)
	26–50	6 (17)
	> 50	6 (17)
Education	Some high school	1 (3)
	High school	4 (11)
	Associate	6 (17)
	Some college	15 (43)
	Finished college	6 (17)
	Advanced degree	3 (9)
Ethnicity	White	18 (51)
	Non-White	17 (49)
Coffee Frequency of Use	Never	10 (29)
	Monthly	4 (11)
	Weekly	8 (23)
	Daily	13 (37)
CBD Use	No	28 (80)
	Yes Monthly Weekly Daily	7 (20) 0 (0) 3 (9) 4 (11)
Marijuana Use	No	19 (54)
	Yes Monthly Weekly Daily	16 (46) 0 (0) 6 (17) 10 (29)
Cigarette Use	No	29 (83)
	Yes	6 (17)

Thirty subjects received CBD during visit 1 while 27 received placebo during visit 1 (Fig. 2). Among the 30 subjects who received CBD during visit 1, seven were lost to follow up and four were excluded from the final analysis for the following reasons: one was excluded due to erroneous administration of CBD during both visits 1 and 2; one was excluded to avoid potential multiple drug interaction because drugs other than THC were detected during urine test; the other two were excluded due to incomplete data. Among the 27 subjects who received placebo during visit 1, six were lost to follow up and five were excluded from the final analysis; three were excluded because drugs other than THC were detected during urine test and the other two were excluded due to incomplete data. Therefore, a total of 35 subjects were included in the final statistical analyses. The large dropout between visits 1 and 2 was due to the COVID-19 pandemic that caused this study to be halted for 6 months due to social distancing measures and subsequent university closure.

### Learning and memory

Table 3 shows the mean scores of the primary trial outcomes (CBD vs. placebo) for the dependent variables measured using the Linear Mixed Models. CBD and placebo groups were not statistically different for the Sum of List A Trials (*P =* 0.47), List B (*P =* 0.78), PI ratio (*P =* 0.17), RI ratio (*P =* 0.38), or FS ratio (*P =* 0.55). Results for total prose recall and MOCA did not also show any statistically significant differences between CBD and placebo (*P =* 0.34 and *P =* 0.63, respectively).

**Table 3:** Mean scores of the primary trial outcomes (CBD vs. placebo) for the dependable variables measured using the Linear mixed models with Bonferroni corrections (*n* = 35)*

Score	CBD Mean [SE]	Placebo Mean [SE]	Adjusted Group Differences	95% CI	*P* Value	DF	F	Effect Size
MOCA	26.63 [0.46]	26.31 [0.46]	0.31	-0.98 to 1.61	0.63	60	0.24	0.06
Sum of Trials A1-A5	47.17 [1.85]	49.09 [1.85]	-1.91	-7.16 to 3.33	0.47	60	0.53	-0.09
List B	6.03 [0.37]	5.89 [0.37]	0.14	-0.89 to 1.18	0.78	60	0.08	0.03
Proactive Interference (PI) Ratio	1.21 [0.08]	1.04 [0.08]	0.17	-0.07 to 0.40	0.17	60	1.98	0.18
Retroactive Interference (RI) ratio	0.83 [0.03]	0.87 [0.03]	-0.04	-0.13 to 0.05	0.38	60	0.78	-0.11
Forgetting Speed (FS) ratio	1.06 [0.03]	1.03 [0.03]	0.03	-0.06 to 0.12	0.55	60	0.36	0.08
Total prose recall	25.49 [1.46]	27.49 [1.46]	-2.00	-6.12 to 2.12	0.34	60	0.95	-0.11

No significant interaction effect was found between the treatment groups (CBD vs. placebo) and history of CBD use. Therefore, data for non-CBD users and frequent CBD users were pooled. No statistically significant differences were observed at the 5% level between non-CBD users and frequent CBD users for any of the variables measured. Among the 28 non-CBD users, 12 (43%) self-reported as marijuana users, and all these 12 subjects also tested positive for urine THC. Among the 7 frequent CBD users, 4 (57%) self-reported as marijuana users and all these 4 also tested positive for urine THC.

The results showed no significant interaction effects between treatment groups (CBD vs. placebo) and urine THC groups (THC positive vs. THC negative). When data for urine THC positive and urine THC negative subjects were pooled, there were no significant effects of urine THC results on CBD’s modulation of L&M at the 5% level. Also, no statistically significant interaction effects between treatment groups (CBD vs. placebo) and sex (male vs. female) were observed for the variables analyzed. Pooling sex data revealed no sex effects at the 5% level for any of the variables measured.

There were, however, highly significant interaction effects between treatment groups (CBD vs. placebo) and age of subjects in the Linear Mixed Model analysis for PI ratio (*P* = 0.008; *n* = 35). Therefore, participants were separated into these two age groups: less < 50 and > 50, because literature reports the start of memory impairment at age 50 (Crook et al. 1990; McEntee and Crook 1990). A t-test was done to compare CBD recipients with placebo recipients within each age group. No treatment effect was observed among younger adults (*P* = 0.36, *n* = 29). Among these 29 adolescents, 15 (52%) tested positive for urine THC. Only one of the 6 adults over 50 tested positive for urine THC. Among adults over 50 years of age, CBD administration did not significantly impact any variables at the significance of *P* < 0.05.

## Discussion

The results of this study suggest that administration of CBD alone does not significantly impair L&M in healthy adults. These findings are consistent with a recent comprehensive review of 73 research papers that concluded that no differences were found between CBD and placebo groups based on various types of assessment of cognitive or psychomotor performance outcomes (Lo et al. 2024). The results of the current study are also consistent with another research that examined the effect of CBD on 60 healthy adults and found that a single dose of 800 mg CBD alone had no impact on the emotional state, cognitive performance, and attention of subjects (Woelfl et al. 2020). Our findings are also consistent with research that showed no modulation by CBD of the cognitive effect of THC when the two drugs were co-administered (Englund et al. 2023). A clinical trial designed to examine the effect of CBD on cannabis use disorder found that CBD administration had no effect on cognitive function (Lees et al. 2023). A recent study examined the acute effects of different types of cannabis on young adult and adolescent resting-state brain networks and found no interactive effects between CBD and THC (Ertl et al. 2024). We did not co-administer CBD and THC during the current trial; however, THC was detected in the urine of the subjects at the time of CBD administration.

In contrast, a memory study conducted using vaping CBD e-liquid (0.25 ml, 5% CBD, 12.5 mg CBD) found that CBD enhanced word recall (Hotz et al. 2021). The discrepancy between the former research and the current one may be due to variations in the protocols and the demographic groups examined. For instance, literature shows that the bioavailability of inhaled THC is at least twice as high as that of oral THC (Chayasirisobhon 2020); therefore, the vaping mode of administration used in the previous study might have resulted in a greater CBD bioavailability and effect in a relatively short time than our trial. In addition, the previous researchers used the urine screen to exclude participants with recent intake of alcohol, CBD, and THC prior to experimentation. For our study, we wanted to examine potential CBD-THC interactions, and we did not exclude urine THC positive participants. The previous study also evaluated the effects of CBD by asking participants to complete free recall only once 20 min after vaping CBD. Our study used CBD press pills and we started our cognitive testing 2 h post oral drug administration. Our study design also allowed for more opportunities for subjects to encode and consolidate over five learning trials, instead of just one recall. In our study, learning was progressive as List A recall increased significantly after each of the five trials. In addition, our RAVLT-R design allowed for the investigation of CBD’s memory protective properties as we were able to examine CBD’s effects on interference during learning. The RAVLT-R measures were used to calculate the PI ratio and RI ratio. To our knowledge, the current research was the first in the literature to quantify CBD’s memory protective qualities via PI ratio and RI ratio.

In addition, the current study involved use of several different learning and memory assessments including the RAVLT-R (assesses verbal declarative memory), Logical Memory Subject of the Wechsler Memory Scale (assesses verbal logical memory), and the MOCA assessment (assesses basal cognitive function). Comparatively, Hotz et al. (2021) utilized only one L&M assessment in which recall was performed only once after vaping drug administration; this is comparable to the first trial recall score from the current study. Thus, the current study was more comprehensive. Although the results from the current study did not find the effects reported by the previous research group, the two studies are similar in the sense that no impairment of L&M was observed in any of them.

The results of demographic factors on CBD’s modulation of human learning and memory were also investigated in this study. As indicated earlier, no significant interaction effects between treatment groups (CBD vs. placebo) and sex were observed during this study for any of the variables analyzed, indicating that sex did not affect the potential effect of CBD on L&M. Previous cognitive studies also found no cannabis by sex interactions (Wade et al. 2024). Although there were reported sex-related differences in memory recall with women outperforming men during some studies (Lowe et al. 2003; Pauls et al. 2013; Sundermann et al. 2016), none of these studies specifically examined the effect of CBD on L&M.

Although the data showed some tendency for CBD administration to result in a higher PI ratio in these older adults (*P* = 0.0505; *n* = 6; effect size = -0.46; Cohen’s d: -1.05; df = 5) compared to when the same subjects received placebo (mean = 1.84, SE = 0.75; mean = 0.96, SE = 0.39; respectively), the number of subjects tested (*n* = 6) is too small to draw any reliable conclusions. Yet, these subjects were being compared to themselves and only one of the 6 adults over age 50 tested positive for urine THC, suggesting a potential intrinsic effect of CBD on L&M in these older subjects. The authors suggest that the possibility of an effect of CBD on L&M in adults over 50 not be disregarded, especially considering that a recent clinical trial also suggested that daily administration of a high CBD dose (800 mg) might improve working memory in people with cannabis use disorder (Lees et al. 2023). Further research involving a higher number of adult participants is required to assess any potential protective memory effect CBD administration might have among older adults.

### Strengths and limitations

The strengths of our study include the fact it is the first comprehensive study to examine the effect of oral CBD administration on L&M, especially including a proactive interference ratio as an end point for analysis. Also, we conducted a two-arm crossover, randomized, double blinded, study in two visits, comparing subjects to themselves. A major advantage of the two-arm crossover design is that variability is reduced, and fewer subjects may be required than in a parallel design. However, a larger sample size targeting specific demographic groups might have provided more statistical power in the study. This study has other limitations. One limitation is that pharmacokinetic (PK) analysis was not done, so the maximum concentration (Cmax) of CBD and its bioavailability are unknown. Another limitation of the study is that a one-time dose of 246 mg CBD was evaluated; further studies would be needed to evaluate the long-term effects of oral CBD consumption on L&M. As detailed earlier under the methods section, the dose used during the current study was based on previous studies where healthy participants were administered doses of CBD varying from 25 mg to 600 mg (Cunha et al. 1980; Solowij et al. 2018; Linares et al. 2019; Hosseini et al. 2021). Yet, there is still the possibility that a one-time 246 mg dosage of CBD may not have been enough to facilitate cognitive effects in this study. For instance, examination of the effect of oral 10 mg THC, 600 mg CBD, or placebo on neural activation during emotional processing found that CBD did decrease the processing of intensely fearful faces 1–2 h after administration compared to placebo (Fusar-Poli et al. 2009).

However, oral administration of as high as 800 mg of CBD did not facilitate a significant physiological response in healthy participants based on several past reports. Specifically, a single dose of 800 mg CBD alone had no impact on the emotional state, cognitive performance, and attention of healthy adults (Woelfl et al. 2020). Another study tested the effects of CBD on impulsivity and memory during abstinence in cigarette dependent smokers and found that “a single 800 mg dose of CBD does not improve verbal or spatial working memory” (Hindocha et al. 2018). A different report found that oral administration of 200–800 mg CBD produced no significant psychoactive or cardiovascular effects in healthy subjects (Haney et al. 2016). These authors also reported that 200–800 mg CBD did not reduce the reinforcing, physiological, or positive subjective effects of smoked cannabis.

## Conclusions

The results of this study suggest three major implications: (a) administration of oral CBD alone did not significantly impair L&M in healthy adults; (b) there were no interaction effects between oral CBD administration and presence of THC; (c) there were highly significant interaction effects between drug treatments and age groups suggesting that adolescents and adults show differential effects of oral CBD; therefore, adolescent CBD users may be at different risk than older adults. There was a trend for potential memory protective effect in older adults; however, considering the low number of adult participants in the current trial, further research involving larger groups is needed to confirm any potential memory protective effects among older adults and test the hypothesis that CBD could be a positive modulator of human L&M through a mechanism of diminished retroactive and proactive interference.

## Electronic supplementary material

Below is the link to the electronic supplementary material.

Supplementary Material 1

## Data Availability

The research participants completed a demographics questionnaire and provided personal information; our subjects were guaranteed that the raw data will remain confidential and will be used according US data privacy laws. Upon reasonable request by an email to moussa.diawara@csupueblo.edu, anonymized data might be shared after the explicit approval by the Colorado State University Institutional Review Board and by the Colorado State University System General Counsel.

## Abbreviations

CBD: Cannabidiol
THC: Tetrahydrocannabinol
MOCA: Montreal Cognitive Assessment
L&M: Learning and Memory
WMS: Wechsler Memory Scale
RAVLT-R: Rey Auditory Verbal Learning Task-Revised
RI: Retroactive Interference
PI: Proactive Interference
FS: Forgetting Speed
