# Supplementary material for: Oral cannabidiol did not impair learning and memory in healthy adults
Source: J Cannabis Res. 2025 Jan 23;7:5. doi: 10.1186/s42238-025-00262-2 (PMC11756171; doi:10.1186/s42238-025-00262-2)
Supplement: Supplementary file 1 — Supplementary Material 1 [file 42238_2025_262_MOESM1_ESM.docx]

LINEAR MIXED MODELS

FACTOR: TREATMENT (CBD vs PLACEBO)

COVARIATES: CBD USE HISTORY, AGE, SEX, URINE THC

INTERACTIONS: TREATMENT*CBD USE HISTORY, TREATMENT*AGE, TREATMENT*SEX, TREATMENT*URINE THC

**Mixed Model Analysis**

| **Case Processing Summary** | | | |
| --- | --- | --- | --- |
|  | | Count | Marginal Percentage |
| TREATMENT | CBD | 35 | 50.0% |
|  | PLACEBO | 35 | 50.0% |
| Valid | | 70 | 100.0% |
| Excluded | | 4 |  |
| Total | | 74 |  |

| **Model Dimension^a^** | | | |
| --- | --- | --- | --- |
|  | | Number of Levels | Number of Parameters |
| Fixed Effects | Intercept | 1 | 1 |
|  | TREATMENT | 2 | 1 |
|  | Sex | 1 | 1 |
|  | CBDUSEHISTORY | 1 | 1 |
|  | URINETHC | 1 | 1 |
|  | AGE | 1 | 1 |
|  | TREATMENT * Sex | 2 | 1 |
|  | TREATMENT * CBDUSEHISTORY | 2 | 1 |
|  | TREATMENT * URINETHC | 2 | 1 |
|  | TREATMENT * AGE | 2 | 1 |
| Residual | |  | 1 |
| Total | | 15 | 11 |
| a. Dependent Variable: MOCA. | | | |

| **Information Criteria^a^** | |
| --- | --- |
| -2 Restricted Log Likelihood | 325.80488945 |
| Akaike's Information Criterion (AIC) | 327.80488945 |
| Hurvich and Tsai's Criterion (AICC) | 327.87385497 |
| Bozdogan's Criterion (CAIC) | 330.89923401 |
| Schwarz's Bayesian Criterion (BIC) | 329.89923401 |
| The information criteria are displayed in smaller-is-better form. | |
| a. Dependent Variable: MOCA. | |

| **Coefficients of Determination** | | |
| --- | --- | --- |
| Pseudo-R Square Measures | Marginal | .114 |
|  | Conditional | .114 |

**Fixed Effects**

| **Type III Tests of Fixed Effects^a^** | | | | |
| --- | --- | --- | --- | --- |
| Source | Numerator df | Denominator df | F | Sig. |
| Intercept | 1 | 60 | 245.162 | <.001 |
| TREATMENT | 1 | 60 | .197 | .659 |
| Sex | 1 | 60 | 1.235 | .271 |
| CBDUSEHISTORY | 1 | 60 | .997 | .322 |
| URINETHC | 1 | 60 | 2.149 | .148 |
| AGE | 1 | 60 | 3.061 | .085 |
| TREATMENT * Sex | 1 | 60 | .487 | .488 |
| TREATMENT * CBDUSEHISTORY | 1 | 60 | 1.244 | .269 |
| TREATMENT * URINETHC | 1 | 60 | .695 | .408 |
| TREATMENT * AGE | 1 | 60.000 | .037 | .849 |
| a. Dependent Variable: MOCA. | | | | |

**Covariance Parameters**

| **Estimates of Covariance Parameters^a^** | | |
| --- | --- | --- |
| Parameter | Estimate | Std. Error |
| Residual | 7.316 | 1.336 |
| a. Dependent Variable: MOCA. | | |

**Estimated Marginal Means**

**TREATMENT**

| **Estimates^a^** | | | | | |
| --- | --- | --- | --- | --- | --- |
| TREATMENT | Mean | Std. Error | df | 95% Confidence Interval | |
|  |  |  |  | Lower Bound | Upper Bound |
| CBD | 26.629^b^ | .457 | 60 | 25.714 | 27.543 |
| PLACEBO | 26.314^b^ | .457 | 60.000 | 25.400 | 27.229 |
| a. Dependent Variable: MOCA. | | | | | |
| b. Covariates appearing in the model are evaluated at the following values: Sex = 1.46, CBD USE HISTORY = .20, URINE THC = .46, AGE = 30.17. | | | | | |

| **Pairwise Comparisons^a^** | | | | | | | |
| --- | --- | --- | --- | --- | --- | --- | --- |
| (I) TREATMENT | (J) TREATMENT | Mean Difference (I-J) | Std. Error | df | Sig.^b^ | 95% Confidence Interval for Difference^b^ | |
|  |  |  |  |  |  | Lower Bound | Upper Bound |
| CBD | PLACEBO | .314 | .647 | 60 | .629 | -.979 | 1.608 |
| PLACEBO | CBD | -.314 | .647 | 60 | .629 | -1.608 | .979 |
| Based on estimated marginal means | | | | | | | |
| a. Dependent Variable: MOCA. | | | | | | | |
| b. Adjustment for multiple comparisons: Least Significant Difference (equivalent to no adjustments). | | | | | | | |

| **Univariate Tests^a^** | | | |
| --- | --- | --- | --- |
| Numerator df | Denominator df | F | Sig. |
| 1 | 60 | .236 | .629 |
| The F tests the effect of TREATMENT. This test is based on the linearly independent pairwise comparisons among the estimated marginal means. | | | |
| a. Dependent Variable: MOCA. | | | |

**Mixed Model Analysis**

| **Case Processing Summary** | | | |
| --- | --- | --- | --- |
|  | | Count | Marginal Percentage |
| TREATMENT | CBD | 35 | 50.0% |
|  | PLACEBO | 35 | 50.0% |
| Valid | | 70 | 100.0% |
| Excluded | | 4 |  |
| Total | | 74 |  |

| **Model Dimension^a^** | | | |
| --- | --- | --- | --- |
|  | | Number of Levels | Number of Parameters |
| Fixed Effects | Intercept | 1 | 1 |
|  | TREATMENT | 2 | 1 |
|  | Sex | 1 | 1 |
|  | CBDUSEHISTORY | 1 | 1 |
|  | URINETHC | 1 | 1 |
|  | AGE | 1 | 1 |
|  | TREATMENT * Sex | 2 | 1 |
|  | TREATMENT * CBDUSEHISTORY | 2 | 1 |
|  | TREATMENT * URINETHC | 2 | 1 |
|  | TREATMENT * AGE | 2 | 1 |
| Residual | |  | 1 |
| Total | | 15 | 11 |
| a. Dependent Variable: Sum of List A Trials . | | | |

| **Information Criteria^a^** | |
| --- | --- |
| -2 Restricted Log Likelihood | 493.81538663 |
| Akaike's Information Criterion (AIC) | 495.81538663 |
| Hurvich and Tsai's Criterion (AICC) | 495.88435215 |
| Bozdogan's Criterion (CAIC) | 498.90973120 |
| Schwarz's Bayesian Criterion (BIC) | 497.90973120 |
| The information criteria are displayed in smaller-is-better form. | |
| a. Dependent Variable: Sum of List A Trials . | |

| **Coefficients of Determination** | | |
| --- | --- | --- |
| Pseudo-R Square Measures | Marginal | .080 |
|  | Conditional | .080 |

**Fixed Effects**

| **Type III Tests of Fixed Effects^a^** | | | | |
| --- | --- | --- | --- | --- |
| Source | Numerator df | Denominator df | F | Sig. |
| Intercept | 1 | 60 | 71.240 | <.001 |
| TREATMENT | 1 | 60.000 | .203 | .654 |
| Sex | 1 | 60 | .545 | .463 |
| CBDUSEHISTORY | 1 | 60 | .236 | .629 |
| URINETHC | 1 | 60 | 1.983 | .164 |
| AGE | 1 | 60.000 | .360 | .551 |
| TREATMENT * Sex | 1 | 60 | .144 | .706 |
| TREATMENT * CBDUSEHISTORY | 1 | 60 | .124 | .726 |
| TREATMENT * URINETHC | 1 | 60 | .013 | .909 |
| TREATMENT * AGE | 1 | 60.000 | .041 | .840 |
| a. Dependent Variable: Sum of List A Trials . | | | | |

**Covariance Parameters**

| **Estimates of Covariance Parameters^a^** | | |
| --- | --- | --- |
| Parameter | Estimate | Std. Error |
| Residual | 120.335 | 21.970 |
| a. Dependent Variable: Sum of List A Trials . | | |

**Estimated Marginal Means**

**TREATMENT**

| **Estimates^a^** | | | | | |
| --- | --- | --- | --- | --- | --- |
| TREATMENT | Mean | Std. Error | df | 95% Confidence Interval | |
|  |  |  |  | Lower Bound | Upper Bound |
| CBD | 47.171^b^ | 1.854 | 60.000 | 43.462 | 50.880 |
| PLACEBO | 49.086^b^ | 1.854 | 60.000 | 45.377 | 52.795 |
| a. Dependent Variable: Sum of List A Trials . | | | | | |
| b. Covariates appearing in the model are evaluated at the following values: Sex = 1.46, CBD USE HISTORY = .20, URINE THC = .46, AGE = 30.17. | | | | | |

| **Pairwise Comparisons^a^** | | | | | | | |
| --- | --- | --- | --- | --- | --- | --- | --- |
| (I) TREATMENT | (J) TREATMENT | Mean Difference (I-J) | Std. Error | df | Sig.^b^ | 95% Confidence Interval for Difference^b^ | |
|  |  |  |  |  |  | Lower Bound | Upper Bound |
| CBD | PLACEBO | -1.914 | 2.622 | 60 | .468 | -7.160 | 3.331 |
| PLACEBO | CBD | 1.914 | 2.622 | 60 | .468 | -3.331 | 7.160 |
| Based on estimated marginal means | | | | | | | |
| a. Dependent Variable: Sum of List A Trials . | | | | | | | |
| b. Adjustment for multiple comparisons: Least Significant Difference (equivalent to no adjustments). | | | | | | | |

| **Univariate Tests^a^** | | | |
| --- | --- | --- | --- |
| Numerator df | Denominator df | F | Sig. |
| 1 | 60 | .533 | .468 |
| The F tests the effect of TREATMENT. This test is based on the linearly independent pairwise comparisons among the estimated marginal means. | | | |
| a. Dependent Variable: Sum of List A Trials . | | | |

**Mixed Model Analysis**

| **Case Processing Summary** | | | |
| --- | --- | --- | --- |
|  | | Count | Marginal Percentage |
| TREATMENT | CBD | 35 | 50.0% |
|  | PLACEBO | 35 | 50.0% |
| Valid | | 70 | 100.0% |
| Excluded | | 4 |  |
| Total | | 74 |  |

| **Model Dimension^a^** | | | |
| --- | --- | --- | --- |
|  | | Number of Levels | Number of Parameters |
| Fixed Effects | Intercept | 1 | 1 |
|  | TREATMENT | 2 | 1 |
|  | Sex | 1 | 1 |
|  | CBDUSEHISTORY | 1 | 1 |
|  | URINETHC | 1 | 1 |
|  | AGE | 1 | 1 |
|  | TREATMENT * Sex | 2 | 1 |
|  | TREATMENT * CBDUSEHISTORY | 2 | 1 |
|  | TREATMENT * URINETHC | 2 | 1 |
|  | TREATMENT * AGE | 2 | 1 |
| Residual | |  | 1 |
| Total | | 15 | 11 |
| a. Dependent Variable: List B Recall. | | | |

| **Information Criteria^a^** | |
| --- | --- |
| -2 Restricted Log Likelihood | 298.80253406 |
| Akaike's Information Criterion (AIC) | 300.80253406 |
| Hurvich and Tsai's Criterion (AICC) | 300.87149957 |
| Bozdogan's Criterion (CAIC) | 303.89687862 |
| Schwarz's Bayesian Criterion (BIC) | 302.89687862 |
| The information criteria are displayed in smaller-is-better form. | |
| a. Dependent Variable: List B Recall. | |

| **Coefficients of Determination** | | |
| --- | --- | --- |
| Pseudo-R Square Measures | Marginal | .044 |
|  | Conditional | .044 |

**Fixed Effects**

| **Type III Tests of Fixed Effects^a^** | | | | |
| --- | --- | --- | --- | --- |
| Source | Numerator df | Denominator df | F | Sig. |
| Intercept | 1 | 60 | 20.123 | <.001 |
| TREATMENT | 1 | 60.000 | .159 | .692 |
| Sex | 1 | 60 | .464 | .498 |
| CBDUSEHISTORY | 1 | 60 | 1.841 | .180 |
| URINETHC | 1 | 60 | .002 | .967 |
| AGE | 1 | 60 | .088 | .768 |
| TREATMENT * Sex | 1 | 60 | .402 | .528 |
| TREATMENT * CBDUSEHISTORY | 1 | 60 | .054 | .817 |
| TREATMENT * URINETHC | 1 | 60 | .090 | .765 |
| TREATMENT * AGE | 1 | 60 | .000 | .995 |
| a. Dependent Variable: List B Recall. | | | | |

**Covariance Parameters**

| **Estimates of Covariance Parameters^a^** | | |
| --- | --- | --- |
| Parameter | Estimate | Std. Error |
| Residual | 4.665 | .852 |
| a. Dependent Variable: List B Recall. | | |

**Estimated Marginal Means**

**TREATMENT**

| **Estimates^a^** | | | | | |
| --- | --- | --- | --- | --- | --- |
| TREATMENT | Mean | Std. Error | df | 95% Confidence Interval | |
|  |  |  |  | Lower Bound | Upper Bound |
| CBD | 6.029^b^ | .365 | 60 | 5.298 | 6.759 |
| PLACEBO | 5.886^b^ | .365 | 60 | 5.155 | 6.616 |
| a. Dependent Variable: List B Recall. | | | | | |
| b. Covariates appearing in the model are evaluated at the following values: Sex = 1.46, CBD USE HISTORY = .20, URINE THC = .46, AGE = 30.17. | | | | | |

| **Pairwise Comparisons^a^** | | | | | | | |
| --- | --- | --- | --- | --- | --- | --- | --- |
| (I) TREATMENT | (J) TREATMENT | Mean Difference (I-J) | Std. Error | df | Sig.^b^ | 95% Confidence Interval for Difference^b^ | |
|  |  |  |  |  |  | Lower Bound | Upper Bound |
| CBD | PLACEBO | .143 | .516 | 60 | .783 | -.890 | 1.176 |
| PLACEBO | CBD | -.143 | .516 | 60 | .783 | -1.176 | .890 |
| Based on estimated marginal means | | | | | | | |
| a. Dependent Variable: List B Recall. | | | | | | | |
| b. Adjustment for multiple comparisons: Least Significant Difference (equivalent to no adjustments). | | | | | | | |

| **Univariate Tests^a^** | | | |
| --- | --- | --- | --- |
| Numerator df | Denominator df | F | Sig. |
| 1 | 60 | .077 | .783 |
| The F tests the effect of TREATMENT. This test is based on the linearly independent pairwise comparisons among the estimated marginal means. | | | |
| a. Dependent Variable: List B Recall. | | | |

**Mixed Model Analysis**

| **Case Processing Summary** | | | |
| --- | --- | --- | --- |
|  | | Count | Marginal Percentage |
| TREATMENT | CBD | 35 | 50.0% |
|  | PLACEBO | 35 | 50.0% |
| Valid | | 70 | 100.0% |
| Excluded | | 4 |  |
| Total | | 74 |  |

| **Model Dimension^a^** | | | |
| --- | --- | --- | --- |
|  | | Number of Levels | Number of Parameters |
| Fixed Effects | Intercept | 1 | 1 |
|  | TREATMENT | 2 | 1 |
|  | Sex | 1 | 1 |
|  | CBDUSEHISTORY | 1 | 1 |
|  | URINETHC | 1 | 1 |
|  | AGE | 1 | 1 |
|  | TREATMENT * Sex | 2 | 1 |
|  | TREATMENT * CBDUSEHISTORY | 2 | 1 |
|  | TREATMENT * URINETHC | 2 | 1 |
|  | TREATMENT * AGE | 2 | 1 |
| Residual | |  | 1 |
| Total | | 15 | 11 |
| a. Dependent Variable: PI ratio (B/A1). | | | |

| **Information Criteria^a^** | |
| --- | --- |
| -2 Restricted Log Likelihood | 122.11073523 |
| Akaike's Information Criterion (AIC) | 124.11073523 |
| Hurvich and Tsai's Criterion (AICC) | 124.17970074 |
| Bozdogan's Criterion (CAIC) | 127.20507979 |
| Schwarz's Bayesian Criterion (BIC) | 126.20507979 |
| The information criteria are displayed in smaller-is-better form. | |
| a. Dependent Variable: PI ratio (B/A1). | |

| **Coefficients of Determination** | | |
| --- | --- | --- |
| Pseudo-R Square Measures | Marginal | .200 |
|  | Conditional | .200 |

**Fixed Effects**

| **Type III Tests of Fixed Effects^a^** | | | | |
| --- | --- | --- | --- | --- |
| Source | Numerator df | Denominator df | F | Sig. |
| Intercept | 1 | 60 | 9.393 | .003 |
| TREATMENT | 1 | 60.000 | 3.564 | .064 |
| Sex | 1 | 60 | .989 | .324 |
| CBDUSEHISTORY | 1 | 60 | .762 | .386 |
| URINETHC | 1 | 60 | 1.590 | .212 |
| AGE | 1 | 60 | 1.760 | .190 |
| TREATMENT * Sex | 1 | 60.000 | 1.056 | .308 |
| TREATMENT * CBDUSEHISTORY | 1 | 60 | 2.228 | .141 |
| TREATMENT * URINETHC | 1 | 60 | 1.189 | .280 |
| TREATMENT * AGE | 1 | 60 | 7.489 | .008 |
| a. Dependent Variable: PI ratio (B/A1). | | | | |

**Covariance Parameters**

| **Estimates of Covariance Parameters^a^** | | |
| --- | --- | --- |
| Parameter | Estimate | Std. Error |
| Residual | .245 | .045 |
| a. Dependent Variable: PI ratio (B/A1). | | |

**Estimated Marginal Means**

**TREATMENT**

| **Estimates^a^** | | | | | |
| --- | --- | --- | --- | --- | --- |
| TREATMENT | Mean | Std. Error | df | 95% Confidence Interval | |
|  |  |  |  | Lower Bound | Upper Bound |
| CBD | 1.208^b^ | .084 | 60.000 | 1.041 | 1.376 |
| PLACEBO | 1.042^b^ | .084 | 60.000 | .874 | 1.209 |
| a. Dependent Variable: PI ratio (B/A1). | | | | | |
| b. Covariates appearing in the model are evaluated at the following values: Sex = 1.46, CBD USE HISTORY = .20, URINE THC = .46, AGE = 30.17. | | | | | |

| **Pairwise Comparisons^a^** | | | | | | | |
| --- | --- | --- | --- | --- | --- | --- | --- |
| (I) TREATMENT | (J) TREATMENT | Mean Difference (I-J) | Std. Error | df | Sig.^b^ | 95% Confidence Interval for Difference^b^ | |
|  |  |  |  |  |  | Lower Bound | Upper Bound |
| CBD | PLACEBO | .167 | .118 | 60 | .165 | -.070 | .403 |
| PLACEBO | CBD | -.167 | .118 | 60 | .165 | -.403 | .070 |
| Based on estimated marginal means | | | | | | | |
| a. Dependent Variable: PI ratio (B/A1). | | | | | | | |
| b. Adjustment for multiple comparisons: Least Significant Difference (equivalent to no adjustments). | | | | | | | |

| **Univariate Tests^a^** | | | |
| --- | --- | --- | --- |
| Numerator df | Denominator df | F | Sig. |
| 1 | 60 | 1.977 | .165 |
| The F tests the effect of TREATMENT. This test is based on the linearly independent pairwise comparisons among the estimated marginal means. | | | |
| a. Dependent Variable: PI ratio (B/A1). | | | |

**Mixed Model Analysis**

| **Case Processing Summary** | | | |
| --- | --- | --- | --- |
|  | | Count | Marginal Percentage |
| TREATMENT | CBD | 35 | 50.0% |
|  | PLACEBO | 35 | 50.0% |
| Valid | | 70 | 100.0% |
| Excluded | | 4 |  |
| Total | | 74 |  |

| **Model Dimension^a^** | | | |
| --- | --- | --- | --- |
|  | | Number of Levels | Number of Parameters |
| Fixed Effects | Intercept | 1 | 1 |
|  | TREATMENT | 2 | 1 |
|  | Sex | 1 | 1 |
|  | CBDUSEHISTORY | 1 | 1 |
|  | URINETHC | 1 | 1 |
|  | AGE | 1 | 1 |
|  | TREATMENT * Sex | 2 | 1 |
|  | TREATMENT * CBDUSEHISTORY | 2 | 1 |
|  | TREATMENT * URINETHC | 2 | 1 |
|  | TREATMENT * AGE | 2 | 1 |
| Residual | |  | 1 |
| Total | | 15 | 11 |
| a. Dependent Variable: RI ratio (A6/A5). | | | |

| **Information Criteria^a^** | |
| --- | --- |
| -2 Restricted Log Likelihood | 5.94811520 |
| Akaike's Information Criterion (AIC) | 7.94811520 |
| Hurvich and Tsai's Criterion (AICC) | 8.01708072 |
| Bozdogan's Criterion (CAIC) | 11.04245977 |
| Schwarz's Bayesian Criterion (BIC) | 10.04245977 |
| The information criteria are displayed in smaller-is-better form. | |
| a. Dependent Variable: RI ratio (A6/A5). | |

| **Coefficients of Determination** | | |
| --- | --- | --- |
| Pseudo-R Square Measures | Marginal | .104 |
|  | Conditional | .104 |

**Fixed Effects**

| **Type III Tests of Fixed Effects^a^** | | | | |
| --- | --- | --- | --- | --- |
| Source | Numerator df | Denominator df | F | Sig. |
| Intercept | 1 | 60 | 53.321 | <.001 |
| TREATMENT | 1 | 60 | .197 | .659 |
| Sex | 1 | 60 | 1.972 | .165 |
| CBDUSEHISTORY | 1 | 60 | .311 | .579 |
| URINETHC | 1 | 60 | .563 | .456 |
| AGE | 1 | 60 | .614 | .436 |
| TREATMENT * Sex | 1 | 60 | .056 | .813 |
| TREATMENT * CBDUSEHISTORY | 1 | 60 | .003 | .960 |
| TREATMENT * URINETHC | 1 | 60 | .120 | .730 |
| TREATMENT * AGE | 1 | 60.000 | .398 | .531 |
| a. Dependent Variable: RI ratio (A6/A5). | | | | |

**Covariance Parameters**

| **Estimates of Covariance Parameters^a^** | | |
| --- | --- | --- |
| Parameter | Estimate | Std. Error |
| Residual | .035 | .006 |
| a. Dependent Variable: RI ratio (A6/A5). | | |

**Estimated Marginal Means**

**TREATMENT**

| **Estimates^a^** | | | | | |
| --- | --- | --- | --- | --- | --- |
| TREATMENT | Mean | Std. Error | df | 95% Confidence Interval | |
|  |  |  |  | Lower Bound | Upper Bound |
| CBD | .828^b^ | .032 | 60.000 | .764 | .891 |
| PLACEBO | .868^b^ | .032 | 60.000 | .804 | .931 |
| a. Dependent Variable: RI ratio (A6/A5). | | | | | |
| b. Covariates appearing in the model are evaluated at the following values: Sex = 1.46, CBD USE HISTORY = .20, URINE THC = .46, AGE = 30.17. | | | | | |

| **Pairwise Comparisons^a^** | | | | | | | |
| --- | --- | --- | --- | --- | --- | --- | --- |
| (I) TREATMENT | (J) TREATMENT | Mean Difference (I-J) | Std. Error | df | Sig.^b^ | 95% Confidence Interval for Difference^b^ | |
|  |  |  |  |  |  | Lower Bound | Upper Bound |
| CBD | PLACEBO | -.040 | .045 | 60 | .379 | -.130 | .050 |
| PLACEBO | CBD | .040 | .045 | 60 | .379 | -.050 | .130 |
| Based on estimated marginal means | | | | | | | |
| a. Dependent Variable: RI ratio (A6/A5). | | | | | | | |
| b. Adjustment for multiple comparisons: Least Significant Difference (equivalent to no adjustments). | | | | | | | |

| **Univariate Tests^a^** | | | |
| --- | --- | --- | --- |
| Numerator df | Denominator df | F | Sig. |
| 1 | 60 | .784 | .379 |
| The F tests the effect of TREATMENT. This test is based on the linearly independent pairwise comparisons among the estimated marginal means. | | | |
| a. Dependent Variable: RI ratio (A6/A5). | | | |

**Mixed Model Analysis**

| **Case Processing Summary** | | | |
| --- | --- | --- | --- |
|  | | Count | Marginal Percentage |
| TREATMENT | CBD | 35 | 50.0% |
|  | PLACEBO | 35 | 50.0% |
| Valid | | 70 | 100.0% |
| Excluded | | 4 |  |
| Total | | 74 |  |

| **Model Dimension^a^** | | | |
| --- | --- | --- | --- |
|  | | Number of Levels | Number of Parameters |
| Fixed Effects | Intercept | 1 | 1 |
|  | TREATMENT | 2 | 1 |
|  | AGE | 1 | 1 |
|  | Sex | 1 | 1 |
|  | CBDUSEHISTORY | 1 | 1 |
|  | URINETHC | 1 | 1 |
|  | TREATMENT * AGE | 2 | 1 |
|  | TREATMENT * Sex | 2 | 1 |
|  | TREATMENT * CBDUSEHISTORY | 2 | 1 |
|  | TREATMENT * URINETHC | 2 | 1 |
| Residual | |  | 1 |
| Total | | 15 | 11 |
| a. Dependent Variable: Forgetting speed (A7/A6). | | | |

| **Information Criteria^a^** | |
| --- | --- |
| -2 Restricted Log Likelihood | 8.23129778 |
| Akaike's Information Criterion (AIC) | 10.23129778 |
| Hurvich and Tsai's Criterion (AICC) | 10.30026329 |
| Bozdogan's Criterion (CAIC) | 13.32564234 |
| Schwarz's Bayesian Criterion (BIC) | 12.32564234 |
| The information criteria are displayed in smaller-is-better form. | |
| a. Dependent Variable: Forgetting speed (A7/A6). | |

| **Coefficients of Determination** | | |
| --- | --- | --- |
| Pseudo-R Square Measures | Marginal | .120 |
|  | Conditional | .120 |

**Fixed Effects**

| **Type III Tests of Fixed Effects^a^** | | | | |
| --- | --- | --- | --- | --- |
| Source | Numerator df | Denominator df | F | Sig. |
| Intercept | 1 | 60 | 103.321 | <.001 |
| TREATMENT | 1 | 60 | .083 | .775 |
| AGE | 1 | 60.000 | 1.412 | .239 |
| Sex | 1 | 60 | 1.355 | .249 |
| CBDUSEHISTORY | 1 | 60 | 2.289 | .136 |
| URINETHC | 1 | 60 | .036 | .850 |
| TREATMENT * AGE | 1 | 60 | .657 | .421 |
| TREATMENT * Sex | 1 | 60.000 | .000 | .988 |
| TREATMENT * CBDUSEHISTORY | 1 | 60 | .578 | .450 |
| TREATMENT * URINETHC | 1 | 60 | .119 | .731 |
| a. Dependent Variable: Forgetting speed (A7/A6). | | | | |

**Covariance Parameters**

| **Estimates of Covariance Parameters^a^** | | |
| --- | --- | --- |
| Parameter | Estimate | Std. Error |
| Residual | .037 | .007 |
| a. Dependent Variable: Forgetting speed (A7/A6). | | |

**Estimated Marginal Means**

**TREATMENT**

| **Estimates^a^** | | | | | |
| --- | --- | --- | --- | --- | --- |
| TREATMENT | Mean | Std. Error | df | 95% Confidence Interval | |
|  |  |  |  | Lower Bound | Upper Bound |
| CBD | 1.061^b^ | .032 | 60.000 | .996 | 1.125 |
| PLACEBO | 1.033^b^ | .032 | 60 | .968 | 1.098 |
| a. Dependent Variable: Forgetting speed (A7/A6). | | | | | |
| b. Covariates appearing in the model are evaluated at the following values: AGE = 30.17, Sex = 1.46, CBD USE HISTORY = .20, URINE THC = .46. | | | | | |

| **Pairwise Comparisons^a^** | | | | | | | |
| --- | --- | --- | --- | --- | --- | --- | --- |
| (I) TREATMENT | (J) TREATMENT | Mean Difference (I-J) | Std. Error | df | Sig.^b^ | 95% Confidence Interval for Difference^b^ | |
|  |  |  |  |  |  | Lower Bound | Upper Bound |
| CBD | PLACEBO | .027 | .046 | 60 | .551 | -.064 | .119 |
| PLACEBO | CBD | -.027 | .046 | 60 | .551 | -.119 | .064 |
| Based on estimated marginal means | | | | | | | |
| a. Dependent Variable: Forgetting speed (A7/A6). | | | | | | | |
| b. Adjustment for multiple comparisons: Least Significant Difference (equivalent to no adjustments). | | | | | | | |

| **Univariate Tests^a^** | | | |
| --- | --- | --- | --- |
| Numerator df | Denominator df | F | Sig. |
| 1 | 60 | .359 | .551 |
| The F tests the effect of TREATMENT. This test is based on the linearly independent pairwise comparisons among the estimated marginal means. | | | |
| a. Dependent Variable: Forgetting speed (A7/A6). | | | |

**Mixed Model Analysis**

| **Case Processing Summary** | | | |
| --- | --- | --- | --- |
|  | | Count | Marginal Percentage |
| TREATMENT | CBD | 35 | 50.0% |
|  | PLACEBO | 35 | 50.0% |
| Valid | | 70 | 100.0% |
| Excluded | | 4 |  |
| Total | | 74 |  |

| **Model Dimension^a^** | | | |
| --- | --- | --- | --- |
|  | | Number of Levels | Number of Parameters |
| Fixed Effects | Intercept | 1 | 1 |
|  | TREATMENT | 2 | 1 |
|  | Sex | 1 | 1 |
|  | CBDUSEHISTORY | 1 | 1 |
|  | URINETHC | 1 | 1 |
|  | AGE | 1 | 1 |
|  | TREATMENT * Sex | 2 | 1 |
|  | TREATMENT * CBDUSEHISTORY | 2 | 1 |
|  | TREATMENT * URINETHC | 2 | 1 |
|  | TREATMENT * AGE | 2 | 1 |
| Residual | |  | 1 |
| Total | | 15 | 11 |
| a. Dependent Variable: TOTAL PROSE RECALL. | | | |

| **Information Criteria^a^** | |
| --- | --- |
| -2 Restricted Log Likelihood | 464.68063891 |
| Akaike's Information Criterion (AIC) | 466.68063891 |
| Hurvich and Tsai's Criterion (AICC) | 466.74960443 |
| Bozdogan's Criterion (CAIC) | 469.77498347 |
| Schwarz's Bayesian Criterion (BIC) | 468.77498347 |
| The information criteria are displayed in smaller-is-better form. | |
| a. Dependent Variable: TOTAL PROSE RECALL. | |

| **Coefficients of Determination** | | |
| --- | --- | --- |
| Pseudo-R Square Measures | Marginal | .074 |
|  | Conditional | .074 |

**Fixed Effects**

| **Type III Tests of Fixed Effects^a^** | | | | |
| --- | --- | --- | --- | --- |
| Source | Numerator df | Denominator df | F | Sig. |
| Intercept | 1 | 60 | 31.140 | <.001 |
| TREATMENT | 1 | 60.000 | .000 | .986 |
| Sex | 1 | 60 | .000 | .982 |
| CBDUSEHISTORY | 1 | 60 | 1.909 | .172 |
| URINETHC | 1 | 60 | .101 | .751 |
| AGE | 1 | 60 | .092 | .763 |
| TREATMENT * Sex | 1 | 60 | .256 | .615 |
| TREATMENT * CBDUSEHISTORY | 1 | 60 | .736 | .394 |
| TREATMENT * URINETHC | 1 | 60 | .000 | .993 |
| TREATMENT * AGE | 1 | 60.000 | .000 | .995 |
| a. Dependent Variable: TOTAL PROSE RECALL. | | | | |

**Covariance Parameters**

| **Estimates of Covariance Parameters^a^** | | |
| --- | --- | --- |
| Parameter | Estimate | Std. Error |
| Residual | 74.047 | 13.519 |
| a. Dependent Variable: TOTAL PROSE RECALL. | | |

**Estimated Marginal Means**

**TREATMENT**

| **Estimates^a^** | | | | | |
| --- | --- | --- | --- | --- | --- |
| TREATMENT | Mean | Std. Error | df | 95% Confidence Interval | |
|  |  |  |  | Lower Bound | Upper Bound |
| CBD | 25.486^b^ | 1.455 | 60 | 22.576 | 28.395 |
| PLACEBO | 27.486^b^ | 1.455 | 60 | 24.576 | 30.395 |
| a. Dependent Variable: TOTAL PROSE RECALL. | | | | | |
| b. Covariates appearing in the model are evaluated at the following values: Sex = 1.46, CBD USE HISTORY = .20, URINE THC = .46, AGE = 30.17. | | | | | |

| **Pairwise Comparisons^a^** | | | | | | | |
| --- | --- | --- | --- | --- | --- | --- | --- |
| (I) TREATMENT | (J) TREATMENT | Mean Difference (I-J) | Std. Error | df | Sig.^b^ | 95% Confidence Interval for Difference^b^ | |
|  |  |  |  |  |  | Lower Bound | Upper Bound |
| CBD | PLACEBO | -2.000 | 2.057 | 60 | .335 | -6.115 | 2.115 |
| PLACEBO | CBD | 2.000 | 2.057 | 60 | .335 | -2.115 | 6.115 |
| Based on estimated marginal means | | | | | | | |
| a. Dependent Variable: TOTAL PROSE RECALL. | | | | | | | |
| b. Adjustment for multiple comparisons: Least Significant Difference (equivalent to no adjustments). | | | | | | | |

| **Univariate Tests^a^** | | | |
| --- | --- | --- | --- |
| Numerator df | Denominator df | F | Sig. |
| 1 | 60 | .945 | .335 |
| The F tests the effect of TREATMENT. This test is based on the linearly independent pairwise comparisons among the estimated marginal means. | | | |
| a. Dependent Variable: TOTAL PROSE RECALL. | | | |
